# Reviewers in 2019

**DOI:** 10.1098/rsob.200071

**Published:** 2020-03-25

**Authors:** Jonathon Pines

**Affiliations:** *Editor-in-Chief*

The editors of *Open Biology* would like to thank all reviewers who contributed their time by refereeing manuscripts submitted throughout 2019. From previous feedback, we know that most reviewers value recognition of their efforts by the journal and through services, such as *Publons*, which is integrated with Open Biology. To provide an opportunity for recognition, we list the names of all reviewers (unless they have opted out). This article is made permanent and citeable by its digital object identifier (DOI). We encourage you to quote your contribution in your applications for tenure and grants or other forms of research assessment. Where you have provided it, we have included your ORCID, so your contribution can be unambiguously assigned to you. The team really do appreciate all the work our reviewers do on behalf of the journal, and we look forward to working with you again during 2020 and beyond.

Mingarro I
Adam R
Alper H
Alsford S
https://orcid.org/0000-0001-8621-4920
Andor N
Aparecida Rainho C
Apostolou E
Archambault V
https://orcid.org/0000-0002-2857-7667
Atkins JF
Avtanski D
Babik W
https://orcid.org/0000-0002-1698-6615
Banerjee S
Banfi A
Barilla D
Bejarano E
Bernard P
Bernstein S
Bird C
Boosani CS
Bridges D
Brown N
http://orcid.org/0000-0002-8958-7017
Bruno S
Bulgakova N
https://orcid.org/0000-0002-3780-8164
Cai D
Cao SS
Carpene C
Caudy A
Cestari I
Chan EHY
Chan K-L
Cheeseman I
https://orcid.org/0000-0002-3829-5612
Chesler L
Chong WC
Christiaens O
Chung SY
Clemons NJ
Colombo M
https://orcid.org/0000-0003-2252-1424
Cortez D
Craig A
Cui B
De Los Santos T
de Marco A
Delattre M
https://orcid.org/0000-0003-1640-0300
Delogu G
Demmig-Adams B
Dixon NE
Doe C
Doetsch P
Duennwald ML
Duhagon MA
Economou T
Escriva H
Ewer J
Fath T
Frasch W
Fry A
Funabiki H
Gaestel M
Gokey J
Goosens V
Grabowska A
Graeme C
Graham A
Guenal I
Guettler S
Gunzl A
Gupta A
Halder G
Han C
Hao N
Harduin-Lepers A
https://orcid.org/0000-0002-1233-3799
Havird J
https://orcid.org/0000-0002-8692-6503
Hehl R
Hellmann N
Hendershot L
Herrmann J
Hu A
Hughes D
Irani S
Jin Z
Johnson T
Karkonen A
Karpel R
Keasling J
Kemp MG
Keysar S
Klein A
Koch JC
Komatsu T
Kong W-J
Kotak S
Krasikova A
Lachke S
Lawrence PA
Lázaro I
Lehner P
Lenhart KF
Leopoldino AM
Levy D
https://orcid.org/0000-0002-7853-3275
Lin Q
https://orcid.org/0000-0002-9916-7761
Llimargas M
Lorenz A
Lorenz R
Mackereth C
MacNeill F
Mankoo B
https://orcid.org/0000-0002-3508-8300
Markstein M
Martin M
Matos J
Matthews M
McDonald M
McInerney G
Michlewski G
Moeendarbary E
Muzumdar M
Nano M
Naqvi AR
Newbold R
Ng L
Niepa T
Ninh VK
Nitschke W
https://orcid.org/0000-0003-2084-3032
Oakley A
Ochsenreiter T
O'Rahilly S
Paijmans J
Palacios IM
Paladino S
https://orcid.org/0000-0001-5332-6774
Paredez A
Pareek TK
Perez-Plasencia C
Pfanner N
Pollak M
Prior K
Pu M
Qi L
Rass U
Rentmeister A
http://orcid.org/0000-0002-3107-4147
Ribeiro C
Richards M
Rigden D
Rivarola M
Rose-John S
Rowan S
https://orcid.org/0000-0002-1123-6743
Sage P
Salazar-Olivo L
Salinas P
Schock F
Sengupta S
Serra F
Sharbrough J
http://orcid.org/0000-0002-3642-1662
Shu Y
Siddiqi N
http://orcid.org/0000-0002-5540-8340
Sidi S
Simeoni L
Sintim H
Smits VAJ
https://orcid.org/0000-0002-9160-4525
Snitkin E
Srinivasan R
Sun Y
Sussman M
Tang H
Tatsuka M
Tavares A
Tee P
Teeri T
Thesleff I
Thomson N
Titorenko VI
Tobimatsu Y
Tomlinson M
Vaidyanathan K
Venturin M
Vladimir K
Wang Q
Weiskopf K
White B
Williams G
Wilson I
Wodrich H
Wood R
Woolley J
Wyres K
Xia J
Yagi H
Yuchi Y
Zhang X

